# Cancer cases detected in the prevention and control service of a private cancer clinic in Peru

**DOI:** 10.1186/s13027-019-0259-0

**Published:** 2019-11-29

**Authors:** José Revilla-López, Andrea Anampa-Guzmán, Luis Casanova Marquez, Katrina Weeks, Suzanne Pollard, Adriel Olórtegui-Yzú, María Ruiz-Velazco, Alba Davila-Edquen, Daniel Castro-Dorer, Juan Wong-Barrenechea, Jossira Abad-Seminario, Pamela Gonzáles-Ramos, Fiorella Rivera-Sandoval, Carlos Carracedo-Gonzáles

**Affiliations:** 1 Cancer Prevention and Control Service, ALIADA Contra el Cáncer, Lima, Peru; 2 Faculty of Medicine, Universidad Nacional Mayor de San Marcos, Lima, Peru; 3 Deparment of Medical Oncology, ALIADA contra el cáncer, Lima, Peru; 4 Department of International Health, Bloomberg School of Public Health, Johns Hopkins University, Baltimore, USA; 5 Department of Preventive Medicine and Public Health, Universidad Nacional Mayor de San Marcos, Lima, Peru; 6 General Medical Council, ALIADA Contra el Cáncer, Lima, Peru

**Keywords:** Oncology, Early detection of cancer, Mass screening, Diagnosis

## Abstract

**Purpose:**

Describe the characteristics of patients seen at the Cancer Prevention and Control Service at a Peruvian private cancer clinic in 2014.

**Patients and methods:**

This retrospective clinical study analyzed the prevalence of 10 cancers and characteristics of patients seen at a private cancer center located in Lima, Peru. The study sample included 7680 adults, and data were collected from de-identified medical records.

**Results:**

The average age of the patients was 44.71 years and 98,82% of them had private insurance. The majority of patients were women (67.69%). Our gross incidence rate of cancer was 35.16 per 100,000 in the Cancer Prevention and Control Service in 2014. Only 0.35% had cancer, and most of those diagnosed with cancer (77.78%) were diagnosed in the early stages, stages I and II. The two most common cancers observed were breast and thyroid cancer.

**Conclusions:**

The high rates of early, rather than late-stage diagnoses at this clinic are dramatically different than national rates. This difference may be because we are analyzing data from a prevention service seeing mainly patients with private insurance as opposed to national data, which consists primarily of patients seen in oncologic services with national insurance.

## Background

Globally, more than 10 million patients have been diagnosed with cancer annually [1]. In Peru, between 2006 and 2011, the Cancer Epidemiological Surveillance Unit reported an average of 18,319 new cancer cases per year [2]. According to the Global Cancer Observatory (GLOBOCAN), the annual number of new cancer cases in Peru in 2012 was 42,846 [3]. Among total cancer cases, the most frequently diagnosed cancers in the Peruvian population were cervical cancer (14.9%), stomach cancer (11.1%) and breast cancer (10.3%). Among men, the most common cancers were prostate cancer (15.1%), stomach cancer (15.1%) and skin cancer (7.8%), whereas, in women, cervical cancer (24.1%), breast cancer (16.6%) and stomach cancer (8.6%) were the most prevalent [3, 4]. In Lima, the capital of Peru, the number of new cases of cancer is 216.9 per 100,000 (based in 2010–2012 cases) [5].

A cancer screening program involves testing an asymptomatic person with an accurate test to identify if they are likely to have the cancer of interest and further investigating to confirm or exclude the presence of cancer. However, to be a valuable service, a screening program must provide more benefits than harms. Such a plan must also use a suitable screening test that can be followed by effective treatment, adequate support, and efficient health services. Cancer screening aims to prevent cancer deaths and improve quality of life by detecting cancers early and adequately treating them [6].

However, despite declining death rates from preventable cancers in high-income countries, mortality rates remain high in middle-income countries like Peru, as cases are often diagnosed late, and treatment remains limited. In low- and middle-income countries, cancer screening coverage is generally low with vast inequalities among socioeconomic groups [7]. Also, these countries often have inadequate resources for addressing cancers, leading to high mortality rates [8].

The Peruvian Government manages oncology services through the Ministry of Health and the public National Institute of Neoplastic Diseases (INEN) and promotes decentralized cancer prevention and control actions by national, regional, and local governments. Since 2013, the government has provided coverage for certain types of cancer to low-income individuals [9]. But the government is not the only entity that offers oncology services; private clinics also offer oncologic care.

In 2015, 59.1% of the Peruvian population had national health insurance; 31.8% had EsSalud, a public social security health entity; 1.1% had army insurance, and 2.9% had private insurance [10].

“ALIADA contra el cáncer” is a private cancer clinic in Lima. It is the referral center for private insurance companies, but its patient population includes both insured and uninsured individuals. 98.88% of the patients had insurance and 1.11% did not have insurance and paid out of pocket. It has two other branches in two cities of the coast and highland of Peru [11]. ALIADA contra el cáncer has a Cancer Prevention and Control Service that offers a package of screening tests to patients for early cancer diagnosis. Appointments for these services are arranged by phone. Around 15,000 people from all over the country receive cancer screening tests in ALIADA contra el cáncer each year [12].

The purpose of this study is to describe the frequency and characteristics of cancer cases detected in the Cancer Prevention and Control Service of the private clinic ALIADA contra el cáncer in 2014. It is essential given that other screening studies focused on the development of screening and immediate treatment of cervical lesions in Amazonian Peru. These studies found that HPV testing was the most efficient method and emphasized in the training of midwives [13–15]. This study aims to provide data on cancer screening in Peru.

Sensitivities for moderate dysplasia or worse were better for VIA (54.9%) and less favourable for HPV and cytology. In this setting, VIA and CC missed the majority of high-grade disease. Overall, HPV testing performed best. VIA gives immediate results, but will require investment in regular training and supervision. Further work is needed to determine whether screened-positive women should all be treated or triaged with a more specific test.

## Methods

### Study design

This is a descriptive study that retrospectively analyzed data from patients that attended the Cancer Prevention and Control Service at ALIADA contra el cáncer from January to December of 2014.

### Study population and setting

The clinic receives patients from throughout the country. 75.4% of patients come from Lima city; 23.32%, from other parts of the country and 1.28%, from an unknown location.

### Methods

Patients that attend the Cancer Prevention and Control Service receive a checkup according to their age, sex, and insurance coverage. There are 35 types of cancer screening packages at different costs. All the packages include physical examination. The screening packages were chosen by the patient according to what their insurance covered. There are 19 packages for women (Table 1) and 10 for men (Table 2).

**Table 1:**
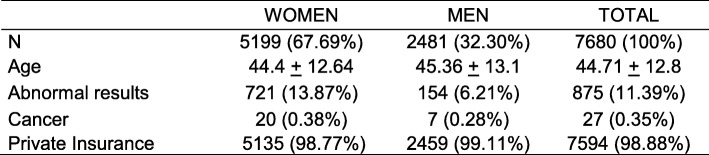
Packages for women

**Table 2:**
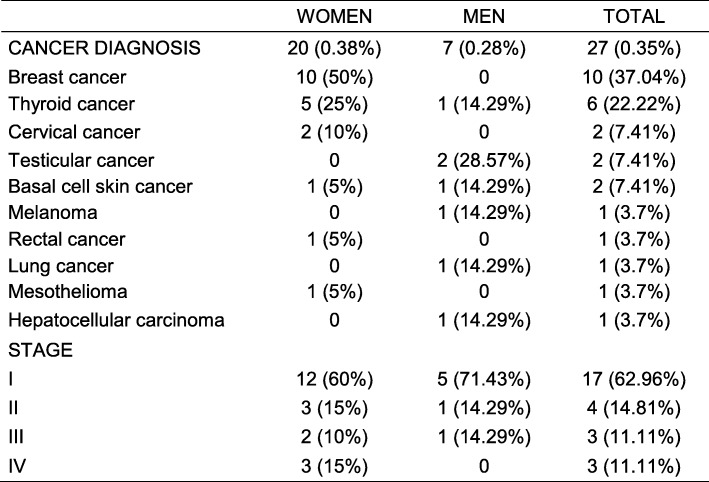
Packaged for men

**Fig. 1:**
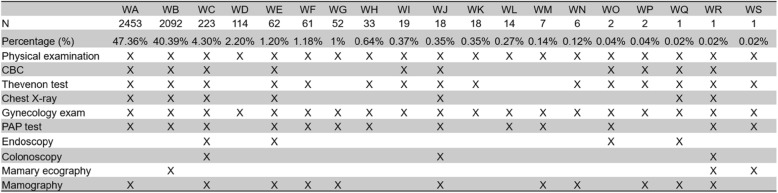
Cancer Screening Algorithm for Cancer Prevention and Control Service at ALIADA

Based on the results of the patient’s examinations, a medical oncologist determines whether there is a suspicion of cancer. If the medical oncologist determines that there is any finding that indicates the likelihood of cancer, a report is personally delivered to the patient. In the absence of a suspicion of cancer, a general practitioner reviews the patient’s result (Fig. 1).

For this study, data were collected from the medical records of all patients that were seen at the Cancer Prevention and Control Service in 2014. The Cancer Prevention and Control Service database was reviewed to identify the age, sex, insurance type, and place of residence of patients who received a cancer diagnosis from the service.

A new case was defined as a person who received a diagnosis of cancer through the pathological sample. Pathological diagnoses were reviewed by the Department of Pathology at the clinic and tumors were classified according to World Health Organization classifications. The TNM classification was used to stage the cancers [16].

### Biostatistical methods

Data are presented as mean ± standard deviation (SD). Data were analyzed using Stata v. 13 (Stata Corp., College Station, TX).

## Results

### Characteristics of the study population

A total of 7680 adult patients were seen, 5199 of whom were women (67.69%). Twenty-eight people did not have their type of package registered (8 men and 20 women). 87.75% of women in the study sample received package WA and WB, two very similar combinations of cancer screenings (Table 1). 88.27% of men that attended the Cancer Prevention and Control Service used two similar screening packages, MA and MB (Table 2).

Table 3 shows the characteristics of patients screened. The mean age of patients was 44 years. Most patients had private insurance coverage. 11.4% of the individuals seen in the Cancer Prevention and Control Service had a screening result that indicated a suspicion of cancer.

**Table 3:** Patient characteristics

	WOMEN	MEN	TOTAL
N	5199 (67.69%)	2481 (32.30%)	7680 (100%)
Age	44.4 ± 12.64	45.36 ± 13.1	44.71 ± 12.8
Abnormal results	721 (13.87%)	154 (6.21%)	875 (11.39%)
Cancer	20 (0.38%)	7 (0.28%)	27 (0.35%)
Private Insurance	5135 (98.77%)	2459 (99.11%)	7594 (98.88%)

### Cancer cases detected in the study population

Of the patients who attended the Cancer Prevention and Control Service in 2014, 0.35% had cancer (Table 4). Our gross incidence rate of cancer was 35.16 per 100,000 (Fig. 1). Most cancer cases (77.78%) were diagnosed in the early stages, I and II. The most common cancer was breast cancer, but the majority were in early stages (six were stage I; two were stage II, and two were stage III). Thyroid cancer was the second most common cancer. Nearly all of these cases were in the early stage (four were stage I; one was stage II, and one was stage III). Surprisingly, there were only two confirmed cases of cervical cancer. One was stage I and the other was stage IV. However, there were 35 premalignant lesions of cervical cancer or NICs (Cervical intraepithelial neoplasia). Thirty were NIC I, the lowest risk to become cervix cancer, and 5 were NIC III, the highest chance to become cervix cancer. Both, testicular and basal cell carcinoma have two stages I cases each. One case of each of the following cancer types was found: melanoma (stage I), rectal cancer (stage II), lung cancer (stage III), mesothelioma (stage IV) and hepatocellular carcinoma (stage I).

**Table 4:** Cancer cases detected

	WOMEN	MEN	TOTAL
CANCER DIAGNOSIS	20 (0.38%)	7 (0.28%)	27 (0.35%)
Breast cancer	10 (50%)	0	10 (37.04%)
Thyroid cancer	5 (25%)	1 (14.29%)	6 (22.22%)
Cervical cancer	2 (10%)	0	2 (7.41%)
Testicular cancer	0	2 (28.57%)	2 (7.41%)
Basal cell skin cancer	1 (5%)	1 (14.29%)	2 (7.41%)
Melanoma	0	1 (14.29%)	1 (3.7%)
Rectal cancer	1 (5%)	0	1 (3.7%)
Lung cancer	0	1 (14.29%)	1 (3.7%)
Mesothelioma	1 (5%)	0	1 (3.7%)
Hepatocellular carcinoma	0	1 (14.29%)	1 (3.7%)
STAGE			
I	12 (60%)	5 (71.43%)	17 (62.96%)
II	3 (15%)	1 (14.29%)	4 (14.81%)
III	2 (10%)	1 (14.29%)	3 (11.11%)
IV	3 (15%)	0	3 (11.11%)

Table 5 shows the characteristics of the patients with cancer at our institution, ALIADA contra el cáncer. All patients with late-stage cancer lived in the capital city. No significant difference was found between the early and late stages groups.

**Table 5:** Characteristics of patients with cancer in ALIADA contra el cáncer according to stage

	EARLY	LATE	TOTAL
N	21 (77.78%)	6 (22.22%)	27 (100%)
AGE	47.81 + 12.36	57.33 + 10.63	49.93 + 12.47
SEX			
Female	15 (55.56%)	5 (18.52%)	20 (74.07%)
Male	6 (22.22%)	1 (3.7%)	7 (25.93%)
LOCATION			
Capital City	18 (85.71%)	6 (100%)	24 (88.89%)
Rest of the country	3 (14.29%)	0	3 (11,11%)
Private Insurance	21 (100%)	6 (100%)	27 (100%)

### Comparison with national data

Table 6 compares the frequency of the types of cancer diagnosed among the study population compared to national data from the INEN [17].

**Table 6:** New cancer cases during 2014 by the institution

	INEN n (%)	ALIADA n (%)
Breast cancer	1214 (10.49%)	10 (37.04%)
Thyroid cancer	564 (4.87%)	6 (22.22%)
Cervix cancer	1484 (12.82%)	2 (7.41%)
Testicular cancer	140 (1.2%)	2 (7.41%)
Basal cell skin cancer	570 (4.92%)	2 (7.41%)
Melanoma	203 (1.75%)	1 (3.7%)
Rectal cancer	223 (1.93%)	1 (3.7%)
Lung cancer	449 (3.88%)	1 (3.7%)
Mesothelioma	–	1 (3.7%)
Hepatocellular carcinoma	212 (1.83%)	1 (3.7%)
TOTAL	11,577 (100%)	27 (100%)

Cervical cancer had the highest percentage among patients seen at INEN in 2014, whereas breast cancer was the most frequently detected type of cancer among patients seen at the Cancer Prevention and Control Service at ALIADA contra el cáncer. The stages at which these cancers were detected also varied drastically between the individuals treated at the private clinic service and the public health insurance system.

Table 7 shows that Across public health facilities in Peru [18], 25% of newly diagnosed cancers were in early stages (stage I-II) and 75% were already in advanced stages (stage III-IV). Meanwhile, the inverse is observed with the individuals in this study. 78% of the cancers diagnosed at the *Aliada* facility in Lima were detected early, and only 22% of cancers were identified in the late stages.

**Table 7:** Distribution of new cancer cases by stage among patients of Seguro Integral de Salud (SIS) and ALIADA contra el cáncer

	^SIS^NHI^a^ n (%)	ALIADA n (%)
EARLY	7461 (24.87%)	21 (77.78%)
Stage I	2441 (8.14%)	17 (62.96%)
Stage II	5020 (16.73%)	4 (14.81%)
LATE	22,539 (75.13%)	6 (22.22%)
Stage III	6148 (20.49%)	3 (11.11%)
Stage IV	16,391 (54.64%)	3 (11.11%)
TOTAL	30,000 (100.00%)	27 (100.00%)

^a^Nationwide data for cancers diagnosed at all National Health Insurance^SIS^public facilities [19]

## Discussion

Since there is not a structured national prevention program in Peru, we compared our results with available data from the oncologic department of the national health insurance, that does not have cancer prevention coverage. Our findings suggest that there are substantial differences in the cancer burden affecting users of Cancer Prevention and Control Service at “ALIADA contra el cáncer” and the public health system in Peru. The distribution of cancer types that were detected among patients who used ALIADA*’s* Cancer Prevention and Control Service was very different from the national data of public facilities in that same year. While geography and the public health system’s larger patient population contribute to these differences, these results demonstrate that the individuals who received cancer care at ALIADA had distinct cancer needs from those who were using the public system. Given these distinctions, strategies to prevent and treat cancer in the public sector may not automatically translate to smaller private facilities. This underscores the importance of using local data to inform cancer prevention and control priorities so that the unique needs of patients in private clinics like ALIADA can be efficiently and adequately met.

The results of this study also indicate that ALIADA’s Cancer Prevention and Control Service was effective in detecting cancers at early stages. An early stage of diagnosis is vital because late-stage cancers are harder to cure, have higher treatment costs, lower quality of life, and a higher likelihood of death. The screening program is aimed to asymptomatic people. The fact that we found advance cancer may be due to the patients ignoring their symptoms. ALIADA’s Cancer Prevention and Control Service managed to detect a much higher proportion of cancers in their early stages than the public insurance health system. While 78% of cancers that were identified at ALIADA were in their early stages, three-quarters of the new cancers detected in the public system were already in advanced stages. This suggests that patients seen at the Cancer Prevention and Control Service are diagnosed in earlier stages than those receiving care from the public insurance system.

Health behaviors and insurance type could explain the high rates of early, rather than late, diagnosis at the Cancer Prevention and Control Service. Individuals who purchased private oncology insurance and attended the Cancer Prevention and Control Service may already be more proactive than the general public in their cancer prevention behaviors. Several studies have also explored the differences in the treatment of cancer according to the type of insurance. It has been argued that private health insurance is preferred by patients because it provides faster diagnosis and treatment [19]. Thus, individuals with existing concerns about their likelihood of cancer who prioritized more rapid diagnosis may have been more likely to self-select into the patient pool at the Cancer Prevention and Control Service.

We compared the characteristics of our patients with early and late stages. Previous studies found that factors associated with a late-stage cancer diagnosis are Afro-American ethnicity, female sex and older age [11, 20]. Even though we did not find any significant difference between the characteristics of patients with early versus late stage cancer, this could be due to the small number of patients with cancer in our sample. Another factor related to late cancer stage diagnosis is the far geographical location from Lima [21, 22]. Surprisingly, all the patients with advanced stage cancer lived in Lima, where the clinic is located. This can be explained by the fact that the majority of patients who come to our clinic are from Lima due to its proximity. Finally, numerous studies indicate that low socioeconomic status, low level of education, and inadequate care seeking behavior were factors associated with late stage diagnosis [22–24]. Unfortunately, this data, which can act as possible confounding factors, were not included in our registries.

Despite the proven benefits of cancer screening, many studies have warned about the possible harms and their underestimation in previous research [25–27]. Cancer screening issues include overdiagnosis and overtreatment. Overdiagnosis is the diagnosis of a condition that would not cause symptoms or death in the patient’s lifetime. Overtreatment refers to therapies that may not be needed and therefore expose patients to adverse effects without a reasonable expectation of benefit [28–30]. These can cause physical and psychosocial harm without any benefit to the patient [31]. The issues and cost-effectiveness of cancer screening are still controversial for these reasons.

The determination of survival time among people who were diagnosed with cancer in an oncologic screening program is challenging due to lead time and length biases. Lead-time bias is a situation in which the early time of diagnosis extends survival even when they do not provide a clinical advantage to the patients. This bias artificially additions survival time to the screen-detected cancer cases [32]. Length bias is another situation when slow-growing tumors that do not develop many symptoms are more likely to be screen-detected. Length bias like lead time bias confers an artificial survival advantage to screen-detected cases [33, 34].

Our study has multiple strengths, as well as some limitations. Even though we had a large sample of patients, our patient sample was predominantly insured by a single sizeable oncologic insurance provider. This can lead to selection bias. Secondly, this study is retrospective as it relies in the limited data in the charts. Thirdly, patient’s received different combinations of screening tests which may result in an underdiagnosis for part of the population. However, all patients were managed in our institution and had a baseline level of engagement with the medical system. Fourthly, chest x-ray is an unapproved modality for lung cancer detection and results related to lung cancer screening detection are hard to interpret. Finally, INEN is the national cancer Institute of Peru to which patients of high complexity are referred for this reason the rate of patients with advanced stages is higher. The comparation with ALIADA contra el cáncer is only for reference.

## Conclusion

To our knowledge, this is the first study that provides data on cancer screening in a Peruvian private health facility. We expect that our data on the most common cancers detected in a private facility will enhance the body of knowledge used by policy-makers to design initiatives on cancer screening programs, enabling these interventions to be more representative and useful for the population of Peru.

Further research on cancer screening programs should also be conducted. A more detailed analysis of the factors associated with early presentation of cancer at ALIADA’s Cancer Prevention and Control Service could allow for the identification of modifiable factors related to the high rate of early detection observed in this program. Such information can help to identify interventions that would be effective in increasing the early detection of cancer throughout Peru. Additionally, future studies could help to define cost-effectiveness measures by including detailed information on the cost of palliative care and the indirect cost of disease-related morbidity and mortality.

## Data Availability

The data that support the findings of this study are available on request from the corresponding author. The data are not publicly available due to privacy or ethical restrictions.
