# Correction to: Cancer cases detected in the prevention and control service of a private cancer clinic in Peru

**DOI:** 10.1186/s13027-019-0267-0

**Published:** 2019-12-19

**Authors:** José Revilla-López, Andrea Anampa-Guzmán, Luis Casanova Marquez, Katrina Weeks, Suzanne Pollard, Adriel Olórtegui-Yzú, María Ruiz-Velazco, Alba Davila-Edquen, Daniel Castro-Dorer, Juan Wong-Barrenechea, Jossira Abad-Seminario, Pamela Gonzáles-Ramos, Fiorella Rivera-Sandoval, Carlos Carracedo-Gonzáles

**Affiliations:** 1 Cancer Prevention and Control Service, ALIADA Contra el Cáncer, Lima, Peru; 2 Faculty of Medicine, Universidad Nacional Mayor de San Marcos, Lima, Peru; 3 Deparment of Medical Oncology, ALIADA contra el cáncer, Lima, Peru; 4 Department of International Health, Bloomberg School of Public Health, Johns Hopkins University, Baltimore, USA; 5 Department of Preventive Medicine and Public Health, Universidad Nacional Mayor de San Marcos, Lima, Peru; 6 General Medical Council, ALIADA Contra el Cáncer, Lima, Peru

**Correction to: Infect Agents Cancer**

**https://doi.org/10.1186/s13027-019-0259-0**

In the original publication of this article [1] several figures and tables were incorrectly displayed. In this correction article the correct overview of the tables and figures are published. The original article has been updated. The publisher apologizes to the readers & authors for the inconvenience.

**Table 1 Packages for women**

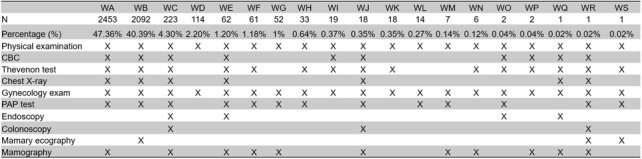



**Table 2 Packages for men**

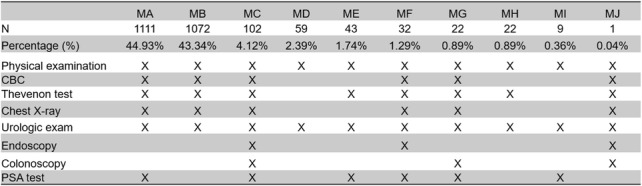



**Figure 1 Cancer Screening Algorithm for Cancer Prevention and Control Service at ALIADA**


